# A user-friendly tool to evaluate the effectiveness of no-take marine reserves

**DOI:** 10.1371/journal.pone.0191821

**Published:** 2018-01-30

**Authors:** Juan Carlos Villaseñor-Derbez, Caio Faro, Melaina Wright, Jael Martínez, Sean Fitzgerald, Stuart Fulton, Maria del Mar Mancha-Cisneros, Gavin McDonald, Fiorenza Micheli, Alvin Suárez, Jorge Torre, Christopher Costello

**Affiliations:** 1 Bren School of Environmental Science and Management, University of California Santa Barbara, Santa Barbara, California, United States of America; 2 Comunidad y Biodiversidad A.C., Calle Isla del Peruano, Guaymas, Sonora, México; 3 School of Life Sciences, Arizona State University, Tempe, Arizona, United States of America; 4 Sustainable Fisheries Group, University of California Santa Barbara, Santa Barbara, California, United States of America; 5 Marine Science Institute, University of California Santa Barbara, Santa Barbara, California, United States of America; 6 Hopkins Marine Station and Center for Ocean Solutions, Stanford University, Pacific Grove, CA, 93950, United States of America; Sveriges lantbruksuniversitet, SWEDEN

## Abstract

Marine reserves are implemented to achieve a variety of objectives, but are seldom rigorously evaluated to determine whether those objectives are met. In the rare cases when evaluations do take place, they typically focus on ecological indicators and ignore other relevant objectives such as socioeconomics and governance. And regardless of the objectives, the diversity of locations, monitoring protocols, and analysis approaches hinder the ability to compare results across case studies. Moreover, analysis and evaluation of reserves is generally conducted by outside researchers, not the reserve managers or users, plausibly thereby hindering effective local management and rapid response to change. We present a framework and tool, called “MAREA”, to overcome these challenges. Its purpose is to evaluate the extent to which any given reserve has achieved its stated objectives. MAREA provides specific guidance on data collection and formatting, and then conducts rigorous causal inference analysis based on data input by the user, providing real-time outputs about the effectiveness of the reserve. MAREA’s ease of use, standardization of state-of-the-art inference methods, and ability to analyze marine reserve effectiveness across ecological, socioeconomic, and governance objectives could dramatically further our understanding and support of effective marine reserve management.

## Introduction

Unsustainable fishing practices threaten biodiversity, conservation, economic and social outcomes [1, 2]. Marine Protected Areas (MPAs; and marine reserves, in which all extractive efforts are prohibited) are frequently proposed to aid in the recovery of fish and invertebrate stocks [3–6] by limiting or restricting fishing effort and gears.

Empirical evidence shows that MPAs increase biomass [4, 7], enhance resilience to climatic impacts [8, 9], and preserve genetic diversity [10]. Compared to MPAs that grant partial protection, marine reserves have higher levels of biomass, density, richness, and larger organisms [3, 11–13]. However, these effects are often measured as biological changes within the reserves through time, and many lack a control site for comparison [14].This approach does not account for other factors (*e.g.* system-level changes in productivity caused by predatory release [15]; or favorable environmental conditions [16]) for which one must control [17] in order to causally attribute a biological change to the reserve. Other studies have used a control-impact comparison approach that uses control sites but does not address temporal variability [4, 7, 18–20].

A smaller fraction of studies have used a before-after-control-impact (*i.e.* BACI) design comparing reserves to control sites before and after implementation [4, 21, 22], which allows the use of causal inference techniques that estimate the effect of the reserve. For example, in ref [21] authors use a BACI design and observe increases in lobster catches –a proxy for abundances– after reserve implementation for protected and control sites. However, the temporal changes in the reserve were greater than in the control site, suggesting a positive effect of the reserve on lobster catches. But even when proper causal inference can be drawn, results are often different across reserves. Effects of reserves on ecological and economic outcomes are highly heterogeneous, and often depend on the specific ecological, economic, and social context.

Standardization of marine reserve evaluation is not new. The IUCN framework “How is your MPA doing?” [23, 24] provides a comprehensive list of biological, socioeconomic, and governance indicators, and insights into how these may be measured or collected. But this framework stops short of analysis, and provides a user with little guidance about establishing causal inference about the reserve. Recent work [25] integrates these three dimensions via the Social Ecological Systems Framework [26, 27] and suggests the use of causal inference techniques to provide a measure of the effect of conservation interventions. However, neither of these approaches provide a user-friendly tool that ensures replicability and scalability of the analysis, particularly when used by the fishers and decision makers themselves.

An increasingly popular way to make science accessible, reproducible, scalable, and replicable is through Open Science and the development of open-access tools [28]. The Ocean Health Index [29, 30], for example, successfully standardized a way to measure the health and benefits of the oceans. This approach has been implemented at a global scale, but also at country-level [31], and regionally [32, 33]. Open access tools are not limited to conservation, and have also been developed to evaluate fishery performance [34, 35], design territorial use rights for fisheries (TURFs, [36]), and improve decision making in the hydro power industry [37].

The purpose of this paper is to describe a user-friendly tool, called “MAREA”, to rigorously systematize the evaluation of marine reserve effectiveness in terms of fisheries and marine conservation goals. The tool is in the form of an open-source application that uses state-of-the-art methods from program evaluation to compare a reserve to control sites along a number of biological, economic, and governance dimensions. We first provide a list of commonly stated management objectives and match them to appropriate indicators. We then develop a simple approach to analyzing these indicators building on causal inference techniques [21], which help us understand the effect of management interventions [25, 38]. To implement the analytical approach, we introduce the Marine Reserve Evaluation Application (MAREA), an open source, web–based tool that automates the framework described in this paper and enables its broader use. Finally, we present a case study on the evaluation of a marine reserve established by the fishers of Isla Natividad (Mexico) in 2006, to demonstrate the potential of MAREA.

## Materials and methods

**Fig 1:**
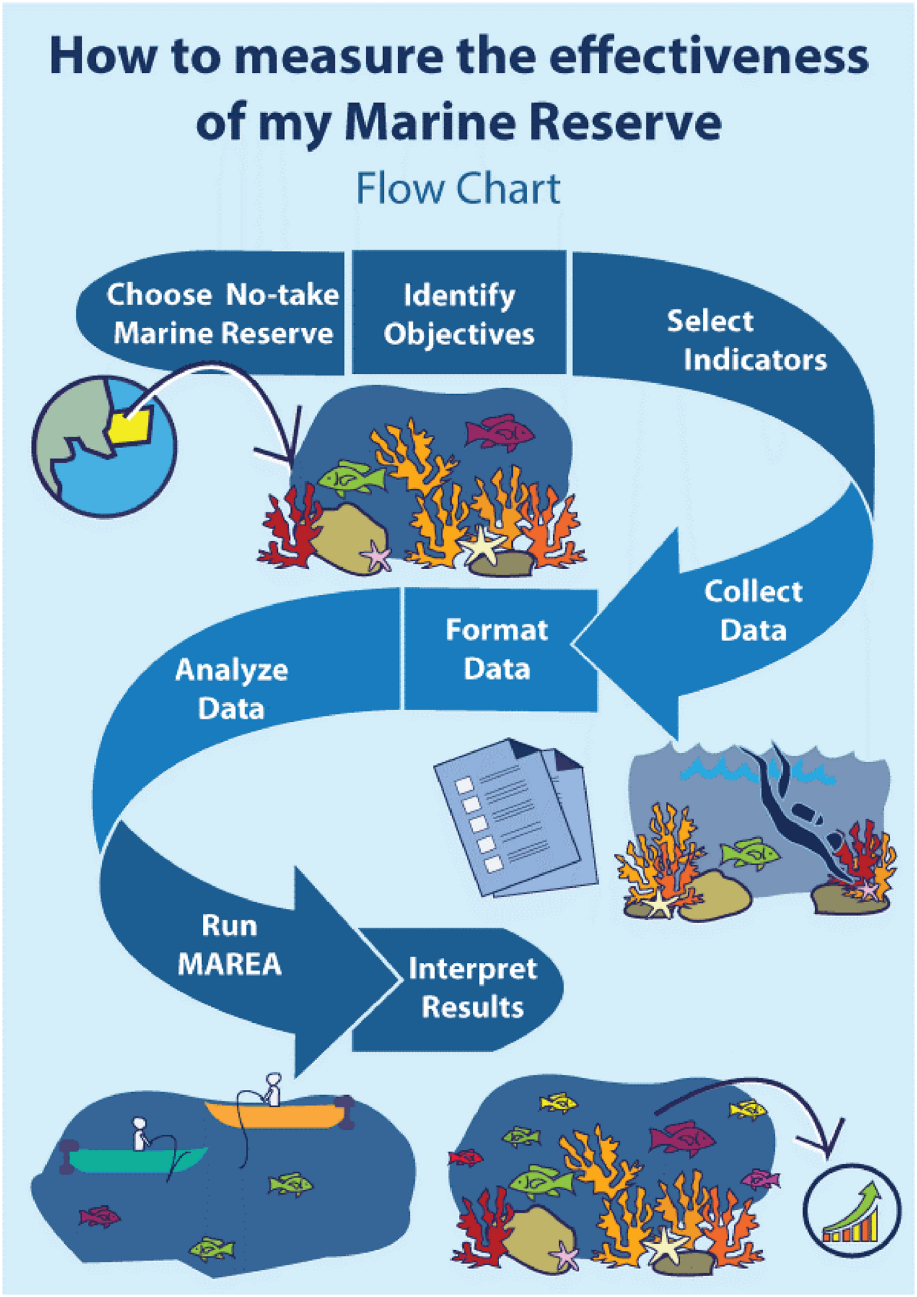
Workflow to evaluate the effectiveness of marine reserves.

Here, we describe the proposed framework to evaluate the effectiveness of marine reserves (Fig 1). We explain how management objectives were identified and matched to appropriate indicators that allow the evaluation of the reserves, and provide brief guidelines on data collection. Alongside, methodologies to analyze these indicators are presented. We then describe the development of MAREA and explain how this tool can be used by fishermen, managers, and other stakeholders with little scientific background. Finally, we provide guidelines on how to interpret and use the results and output generated by MAREA to inform management.

### Marine reserve objectives and indicators

Throughout this study, we will refer to the stated goals for which a marine reserve was designed as “objectives.” This work was motivated by the case of Mexico, where 39 reserves have been implemented over the past five years to achieve objectives such as increasing productivity in nearby waters or recovery of overexploited species; most of these reserves have never been formally evaluated for effectiveness at meeting those objectives. Thus, our focus was on identifying common objectives of marine reserves in Mexico. However, a literature review and discussions with marine reserve researchers suggested that the objectives driving Mexican marine reserve implementation are similar to those in the rest of the world. Thus, we group these objectives into seven major categories that may be applied to marine reserves worldwide. Any given reserve may have been implemented to meet one or more of these. The list includes objectives stated in legislation [39, 40] and official documents such as the Technical Justification Studies (*Estudios Técnicos Justificativos*), agreements, and decrees associated to these areas:

1. Avoid overexploitation
2. Conserve species under a special protection regime
3. Maintain biological processes (reproduction, recruitment, growth, feeding)
4. Improve fishery production in adjacent waters
5. Preserve biological diversity and the ecosystem
6. Recover overexploited species
7. Recover species of economic interest

Based on these seven objectives, we determined a set of associated indicators to evaluate reserve effectiveness. These indicators are specific variables on which data could be collected and analyzed, to ultimately determine whether the corresponding objective was causally being achieved by the marine reserve. The list of indicators was compiled through a review of scientific literature in which we identified indicators that were used to measure similar objectives [3–5, 7, 11, 13, 14, 18–21, 23, 24, 41–44]. A first filter eliminated indicators for which baseline data do not typically exist in Mexico. The preliminary list of indicators was reviewed at a workshop with participation of members from Mexican fishery management agencies and non-government organizations. Later, these were presented to fishers from the Ensenada Fishing Cooperative (*S.C.P.P. Ensenada*), in El Rosario, Baja California, who provided input. Our final list of indicators includes those identified in review works [4, 44].

Indicators are divided into three main categories: biological, socioeconomic, and governance (Table 1). The nine biological indicators focus on fish and invertebrate communities that are evaluated using underwater ecological surveys performed inside and outside the reserve (see Data and Analysis section for specific sampling design and methodologies). Five socioeconomic indicators reflect the performance of the fishery in terms of landings, income from landings, and availability of alternative livelihoods. Fifteen governance indicators describe the governance structures under which the community operates (*e.g.*, access rights to the fishery, number of fishers, legal recognition of the reserve). Most biological and socioeconomic indicators are quantitative and require a numerical entry (*e.g.* Fish biomass) while all governance indicators, one biological indicator, and one socioeconomic indicator are qualitative and rely on a descriptive entry (*e.g.* Reasoning for reserve location). Many of them specifically measure an outcome of the reserve, though some are designed to further the understanding of the mechanisms driving a reserve’s performance. In that sense, most biological and socioeconomic indicators are outcome variables. On the other hand, governance indicators are viewed as possible explanatory variables of reserve performance. Whenever an indicator is applied to “Target species”, it means that the indicator can be used for all species (*e.g.* Fish Biomass) and/or for individual species that are either the conservation target of the reserve or are of particular economic or ecological interest (*e.g.* Grouper Biomass). Finally, indicators B3 and B4 are different in that B3 only looks at the density of organisms above size at first maturity (related to reproductive potential), while B4 measures the density of all fish or of a target species. Each indicator targets different plausible desired outcomes, like increased reproductive potential (*i.e.* B3; [45]) or having more fish -regardless of their size- to attract tourism (*i.e.* B4). Table 1 presents the proposed indicators, and Table 2 shows how objectives are matched with biological and socioeconomic indicators. Governance indicators are excluded from Table 2, but should be considered for every objective as each serves as a plausible explanatory variable for reserve performance.

**Table 1:** List of indicators to evaluate the effectiveness of no-take marine reserves.

Code	Indicator	Data type	Unit
**Biological**			
B1	Shannon diversity index	Continuous	
B2	Species richness	Discrete	Number of species/transect
B3	Density of mature organisms	Continuous	Percent
B4	Density*	Continuous	Organisms/transect
B5	Natural Disturbance	Descriptive	
B6	Mean Trophic Level	Continuous	
B7	Biomass*	Continuous	kg/transect
**Socioeconomic**			
S1	Total landings*	Continuous	kg
S2	Income from total landings*	Continuous	$
S3	Alternative economic opportunities	Ordinal	
**Governance**			
G1	Access to the fishery	Categorical	
G2	Number of fishers	Discrete	
G3	Legal recognition of reserve	Binary	
G4	Reserve type	Descriptive	
G5	Illegal harvesting	Ordinal	
G6	Management plan	Binary	
G7	Reserve enforcement	Descriptive	
G8	Size of reserve	Discrete	
G9	Reasoning for reserve location	Descriptive	
G10	Membership to fisher organizations	Binary	
G11	Type of fisheries organizations	Categorical	
G12	Representation	Ordinal	
G13	Internal Regulation	Binary	
G14	Perceived Effectiveness	Categorical	
G15	Social Impact of Reserve	Categorical	

* Indicates the indicator is applied to target species

**Table 2:** Management objectives and respective performance indicators.

Objective	B1	B2	B3	B4	B4*	B5	B6	B7	B7*	S1	S1*	S2	S2*	S3
Avoid overexploitation			x	x	x	x	x	x	x	x	x	x	x	x
Conserve species under a special protection			x		x	x			x	x		x		x
Maintain biological process	x	x		x		x	x	x						x
Improve fishery production in nearby waters				x	x	x		x	x	x	x	x	x	x
Preserve biological diversity and the ecosystem	x	x		x		x	x	x						x
Recover overexploited species			x		x	x			x		x		x	x
Recover species of economic interest			x		x	x			x		x		x	x

Governance indicators are excluded from the table, but all should be used for any objective.

* Indicates the indicator is applied to target species

### Data and analyses

In many coastal marine reserves, biological data are often collected via underwater visual censuses as part of a reserve’s monitoring program. Scientific divers record fish and invertebrate richness and abundances, as well as fish total length along belt transects. Ecological surveys are typically performed annually in each reserve and corresponding control site(s), before and after the implementation of the reserve, providing a sampling design that can be used to draw causal inference. Control sites are areas where habitat is similar to that of the reserve, but with presence of fishing activity; in principle these are areas that are otherwise observationally identical to the reserve site, but where, for presumably random reasons, a reserve was not implemented. While transect dimensions (*i.e.* length and width) and sampling methods might vary from study to study, the general idea remains the same: richness, abundances, and sizes of organisms are recorded in a study–specific standardized way. For this reason, MAREA does not assume specific transect dimensions, and pertinent indicators are calculated per transect (Table 1). More information on data collection and formatting is provided in a guidebook [46], which is available in English and Spanish in MAREA’s interface.

This sampling design for biological data allows us to use causal inference techniques [21, 47] to evaluate the effect of the reserve on biological indicators. The hypothesis that the indicators will respond to implementation of the reserve is tested by analyzing spatial and temporal changes in each numeric biological indicator (all but B5) using generalized linear models [21]. To account for variations in the environment and survey conditions, covariates that are gathered during the underwater ecological surveys are included in the difference-in-differences model with form:

$$\begin{array}{c} I_{i,t,z}=\beta_{0}+\sum_{t=2}^{T}\gamma_{t}Y_{t}+\beta_{1}Z_{i,z}+\beta_{2}P_{i,t}\times Z_{i,z}+\beta_{3}T_{i,t,z}+\beta_{4}V_{i,t,z}+\beta_{5}D_{i,t,z}+\epsilon_{i,t,z} \end{array}$$ (1)

In this model, *i*, *t*, and *z* are indices for transect, time, and zone (control or reserve site), respectively. This model allows us to estimate the change in an indicator (*I*) based on the year (*Y*), a dummy variable that indicates treatment (*Z*; *i.e.* control or reserve), an interaction between a dummy variable that indicates before or after implementation (*P*) and treatment (*Z*), and covariates such as bottom temperature (*T*; in °C), horizontal visibility during the survey (*V*; in m), and depth at which survey was performed (*D*; in m). *ϵ* represents the error term associated to the regression. Here, years are modeled as factors, using the first year as the reference level. This does not impose a linear structure in the way an indicator changes through time (*i.e.* the change in biomass between 2006 and 2007 does not have to be the same as the change between 2015 and 2016). The treatment and implementation variables, modeled as dummy variables, are coded as Control = 0 and Reserve = 1; and Before implementation = 0 and After implementation = 1, respectively.

Socioeconomic data are often collected by fishers, natural resource management agencies, or Civil Society Organizations (CSOs) by recording landings, income, and sometimes prices for each species. To control for inflation, income is adjusted with the country’s consumer price index [48]:

$$\begin{array}{c} I_{t}=RI\times \frac{CPI_{t}}{CPI_{T}} \end{array}$$ (2)

Where *I*_*t*_ represents the adjusted income for year *t* as the product between the reported income for that year and the ratio between the consumer price index (*CPI*) in that year to the most recent year’s (*T*) CPI. Since no control sites are typically available for this data type, numeric socioeconomic indicators (S1 and S2) are evaluated with a simplified version of Eq (1):

$$\begin{array}{c} I_{t}=\beta_{0}+\beta_{1}P_{t}+\epsilon_{t} \end{array}$$ (3)

This model does not formally allow for causal inference, but we can still measure changes in mean landings and income before and after the implementation of the reserve and provide valuable input. For both models (Eqs (1) and (3)), we estimate the model coefficients with ordinary least squares, and calculate heteroskedastic–robust standard errors [49].

While biological and some economic data are regularly collected, governance data are typically not available nor systematically collected by the community or other organizations. Therefore, we created a survey specifically designed to collect information needed for the proposed indicators (B5, S3, and G1–G15). The survey is included as supplementary material in English (S1 Appendix) and Spanish (S2 Appendix). To analyze governance information, we developed a framework based on a literature review of common governance structures and their relation to effectiveness in managing fisheries or marine reserves (S1 Table). This approach has been proven to successfully evaluate governance structures [50]. Unlike with biological and socioeconomic objectives (see Eqs (1) and (3)), MAREA does not quantitatively analyze governance information. Rather, it is presented along with the biological and socioeconomic indicators to provide managers and users with a more complete description of the reserve.

### Marine Reserve Evaluation App (MAREA)

We developed MAREA in R version 3.4.2 and R Studio 1.1.383 [51] using the Shiny package [52], to build an interactive web application hosted on an open server; the MAREA app can be accessed at turfeffect.shinyapps.io/marea. While the original version was developed in Spanish because it was aimed for Mexico and other Latin-American countries, all of its content can be translated by a translation widget available within the app.

MAREA is designed as a 6-step process, divided in tabs that appear upon launching the app. The first tab introduces the app and summarizes the evaluation process. Then, the user selects management objectives, which MAREA automatically matches to appropriate indicators, based on Table 2. Users can also select and deselect indicators based on their interests and data availability by “clicking” on the check-boxes in MAREA. The user can then load data on one or more reserves, using standard *.csv text files; sample datasets are provided within MAREA. Once data have been loaded, MAREA identifies all reserves in the data, and lets the user select the reserve to be evaluated. At this point, the user can also specify the year of implementation of the reserve, reserve dimensions, and indicate target species that are of particular management interest. MAREA provides the user with a section to confirm that all the decisions made leading up to that point are correct. Once the user has confirmed all input data, objectives, and other information, MAREA performs the formal program evaluation analyses discussed above. For a typical data set, the automated analysis step takes less than one second. Finally, the user is taken to the results tab where all results are presented in a simple format. The user can also download a more comprehensive technical report produced in *.pdf format.

**Fig 2:**
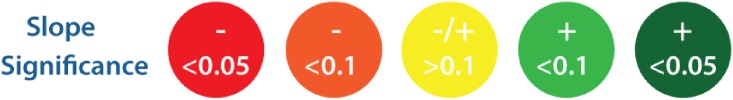
Legend used to interpret the scorecard produced by MAREA. Colors indicate direction of change (red = negative; green = positive), and color intensity is given by the statistical significance.

The first output is a color–coded scorecard intended to provide a general overview of the effectiveness of the reserve. The scorecard provides a global score for the reserve, a general score for each category of indicators, and an individual score for each indicator. The global and category–level scores are determined by the percentage of positive indicators, overall and for each category, respectively. For numeric biological indicators (all but B5), the color is defined by the sign of the interaction term coefficient (*β*_2_) in Eq (1). For socioeconomic indicators, colors are assigned based on the direction of the slope (*β*_1_) in Eq (3). Red, yellow, and green are used for *β*_*i*_ < 0, *β*_*i*_ = 0, and *β*_*i*_ > 0, respectively. The intensity of the color is defined by the significance of the coefficient, testing the null hypothesis of no change (*i.e.* *H*_0_: *β*_*i*_ = 0) with a Student’s t-test. Cutoff values are *p* < 0.05 and *p* < 0.1. Thus, even in a case where *β*_*i*_ > 0, if the coefficient is not significant by standard measures (*i.e.* *p* > 0.1), the indicator will be assigned a yellow color. A legend (Fig 2) is provided within the scorecard to aid in the interpretation of these results. Governance indicators are represented simply by red or green. The color is defined based on what literature shows to be a negative (red) or positive (green) factor for a reserve (S1 Table). For example, if the perceived degree of illegal fishing is high, this indicator will be assigned a red color. However, due to the nature of some governance indicators, which require the user to provide a narrative, only some indicators are presented in the scorecard (although all are included in the technical report).

The second output from MAREA is a technical report intended to communicate information and statistical results in a more comprehensive and technical way. This report also includes a scorecard as a summary of the results, but provides more information for each indicator. For all numeric biological indicators, the report includes a graph of the value of the indicator in the reserve and control sites through time. It also provides a regression table that summarizes the value of all coefficients in the regression and their respective robust standard errors. The summary table also provides information on model fit (*R*^2^) and significance of the regression.

The scorecard is produced with functions from the Shinydashboard package [53]. The technical report is produced by a parameterized Rmarkdown document [54] processed by the knitr package [55]. Another feature of MAREA is that the user can choose to share the data. Once the technical report is downloaded, the information on the reserve, its management objectives, and all uploaded data are saved into a central repository. These data can be accessed at any time by any person interested in acquiring them at github.com/turfeffect/MAREAdata.

### Case study

**Fig 3:**
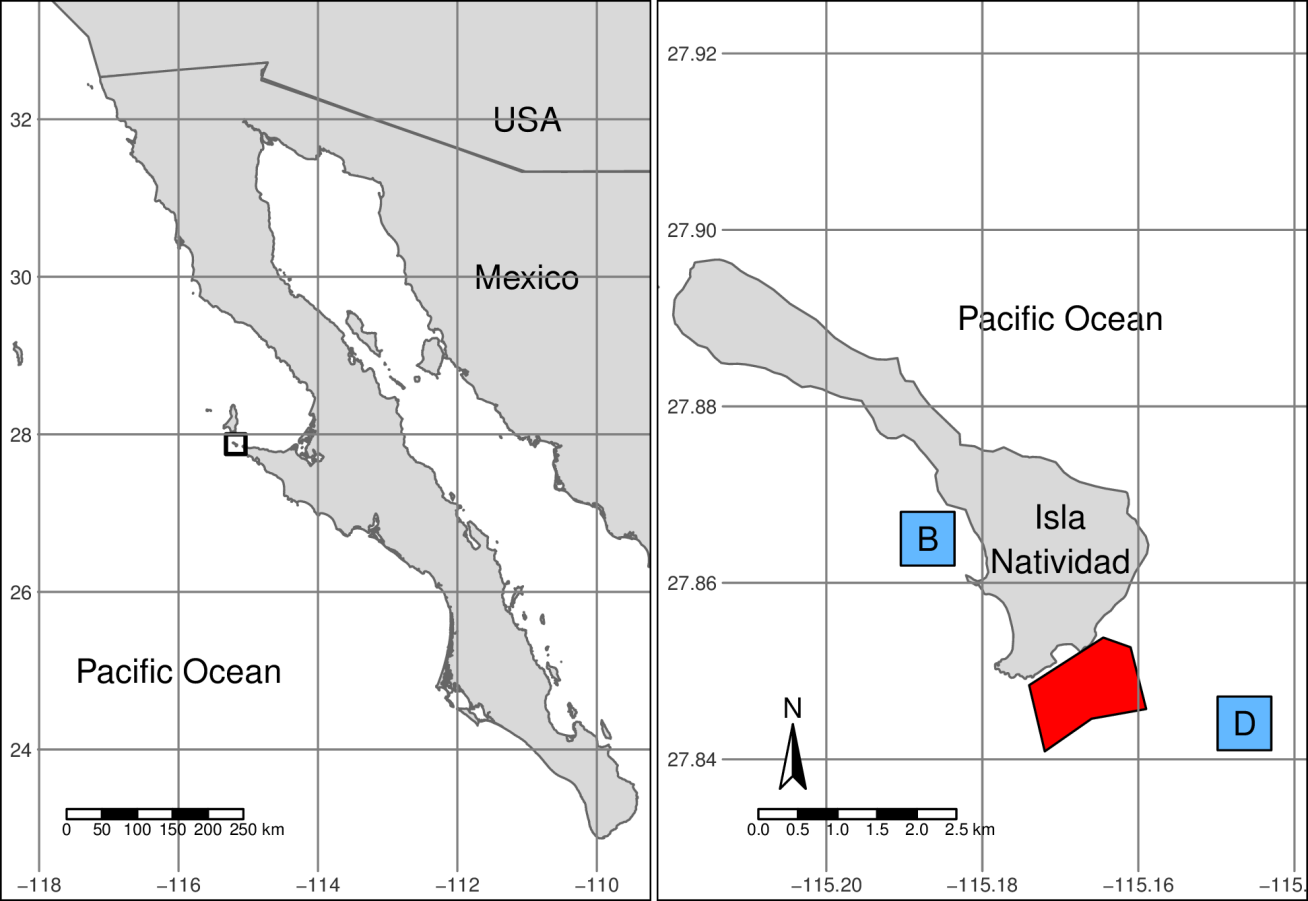
General location of Isla Natividad (left) and map of the island (right). The marine reserve polygon is indicated in red, and the approximate location of control sites is indicated by blue squares (B = Babencho, D = La Dulce). Shapefiles for Mexican coastline and the United States were obtained from INEGI [58] and the tmap R package [59], respectively.

While MAREA is a general tool that can be easily employed to evaluate the effectiveness of any marine reserve with the required input data, we illustrate its use here by applying it to one marine reserve near Isla Natividad, in Baja California Sur, Mexico. Isla Natividad is located 8 Km off the Pacific Coast of the Baja California Peninsula (Fig 3), where fishers operate under a fishing cooperative (*S.C.P.P. Buzos y Pescadores de la Baja California*) that promotes co-management of marine resources [56, 57]. Additionally, fishers have Territorial Use Rights for Fisheries (TURFs) that provide them with exclusive access rights to exploit the benthic marine resources within a given perimeter [57].

In 2006, the Isla Natividad community established a biological baseline following the data collection protocol described in this study. The community then implemented two community-based marine reserves within their TURF [8, 41, 60] after establishing a baseline for the soon-to-be reserves and control sites. Evidence suggest that these reserves have been effective at enhancing resilience to climate variations [8] and preserving genetic diversity of high value commercial species such as abalone [10]. These ecological benefits have been translated into economic benefits, enhancing population persistence and bolstering abalone fisheries [43]. For the purpose of this evaluation, we focused on the “La Plana / Las Cuevas” marine reserve, located at the southern end of the island (Fig 3) and its corresponding control site “La Dulce / Babencho”.

The objective of this reserve was to recover species of economic interest –which were overexploited– and to enhance fishery production in nearby waters. Fishers were also interested in preserving biological diversity and the ecosystem. Thus, objectives 4—7 were selected. Using Table 2 to match these objectives with appropriate management indicators, we selected all biological, socioeconomic, and governance indicators included as options in the framework.

Local fishers (who were trained in scientific diving by the CSO Comunidad y Biodiversidad, A.C. (COBI; www.cobi.org), ReefCheck California, and Stanford University) and personnel from these institutions performed SCUBA dives to record fish and invertebrate richness and abundances, as well as fish total length. They recorded information along 30 m transects, with a sampling window of 2 m x 2 m following a standardized ReefCheck protocol [61]. Ecological surveys were performed yearly in each reserve and corresponding control site(s), before and after the implementation of the reserve, providing the requisite time series data inside the reserve and for a suitable control site. Annual surveys (2006–2016) were carried out in late July—early August, performing a total of 242 and 245 transects in the reserve site for fish and invertebrate surveys, respectively. Similar sampling effort was applied to the control site, with 221 fish and 222 invertebrate transects. Between 12 and 27 transects were performed in each site every year.

Socioeconomic data were obtained from the National Commission for Aquaculture and Fisheries (*Comisión Nacional de Acuacultura y Pesca*; CONAPESCA). The data contains species-level information on monthly landings and income from nine species from 2000 to 2014. Data on landings and income were aggregated by year and species, and adjusted by the Consumer Price Index [48]. From the nine species available, we selected as objective species those that contributed the most (88.27%) income from 2000 to 2014: lobster (*Panulirus interruptus*; 71.76%), red sea urchin (*Mesocentrotus franciscanus*; 9.33%), snail (*Megastraea undosa*; 3.93%), and sea cucumber (*Parastichopus parvimensis*; 3.23%). Abalone species (*Haliotis fulgens*; 4.52% and *Haliotis corrugata*; 6.16%) were excluded because the cooperative implemented an informal closure of these fisheries in 2010 to allow the population to recover. Eliminating all fishing pressure on abalones means that the control site receives (for this species) the same treatment as the reserve.

We constructed the governance data based on local knowledge of the area and the community.

## Results from illustrative example

In this section we show the results of the application of MAREA to the La Plana/Las Cuevas marine reserve in Isla Natividad, Mexico. These results are intended to highlight the relevance and utility of the MAREA framework and app, which automate the analysis and make it replicable. While we highlight some of the general observed trends, we focus on the utility of the tool rather than on the specific effectiveness of this case study marine reserve.

**Fig 4:**
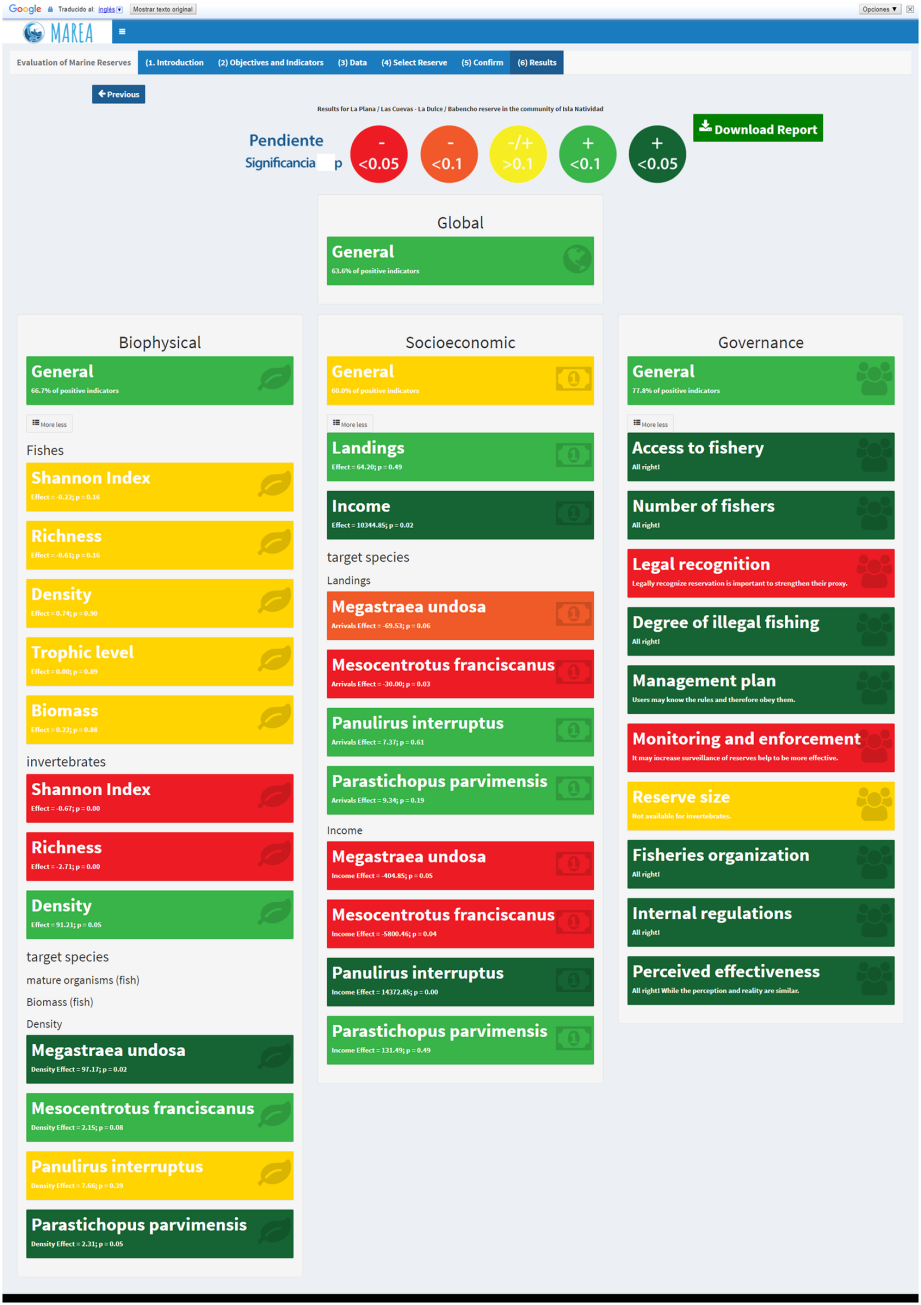
Scorecard produced by MAREA for the “La Plana / Las Cuevas” marine reserve in Isla Natividad, Mexico.

The scorecard (Fig 4) shows that this reserve achieves a general score of 64%, suggesting that 64% of all indicators are positive. All category–level scores were also high, with values of 67%, 60%, and 71% positive indicators for biological, socioeconomic and governance, respectively.

**Fig 5:**
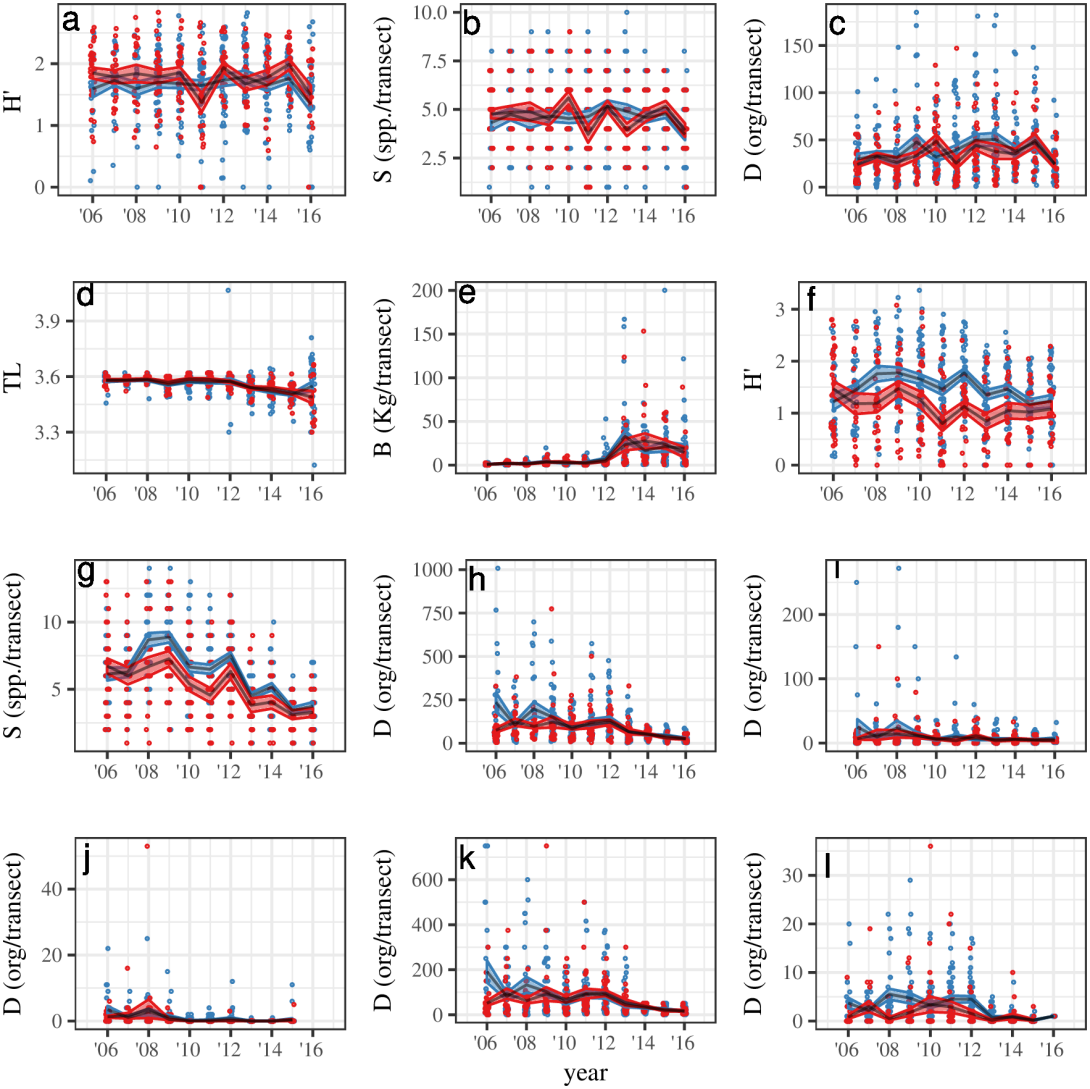
Plots for values of each biological indicator (y-axis) through time (x-axis). Red and blue correspond to the reserve and control sites, respectively. Black lines indicate yearly mean values, and ribbons indicate ± 1 standard error. Dots are horizontally jittered to aid visualization. This figure contains information for fish Shannon’s diversity index (a), fish species richness (b), fish density (c), fish trophic level (d), fish biomass (e), invertebrate Shannon’s diversity index (f), invertebrate species richness (g), invertebrate density (h), lobster density (i), urchin density (j), snail density (k), and sea cucumber density (l).

Among the biological indicators, the greatest effect of the reserve was observed for snail and sea cucumber densities, with values of *β*_2_ = 97.17 (*p* < 0.05) and *β*_2_ = 2.31 (*p* < 0.05), respectively. Fish indicators showed no significant change (*p* > 0.1), with negative trends for Shannon’s diversity index and fish species richness and positive trends for density, biomass, and mean trophic level. Changes through time for these indicators are presented in Fig 5, and a summary of *β*_2_ coefficients is provided in Table 3.

**Table 3:** Summary of average treatment effect of the reserve on biological indicators.

Indicator	Estimate (SD)	t-score
Shannon fish	-0.22 (0.16)	-1.3969
Richness fish	-0.61 (0.43)	-1.4073
Density fish	0.74 (6.15)	0.1205
Trophic fish	0.00 (0.01)	0.1399
Biomass fish	0.22 (1.47)	0.1476
Shannon invert	-0.67 (0.22)**	-3.0481
Richness invert	-2.71 (0.81)**	-3.3519
Density invert	91.21 (47.11)*	1.9362
Lobster	7.66 (8.93)	0.8583
Urchin	2.15 (1.23)*	1.7425
Snail	97.17 (42.90)**	2.2652
Cucumber	2.31 (1.17)**	1.9782

* Indicate significance level, with (*) indicating p < 0.1 and (**) p < 0.05.

**Fig 6:**
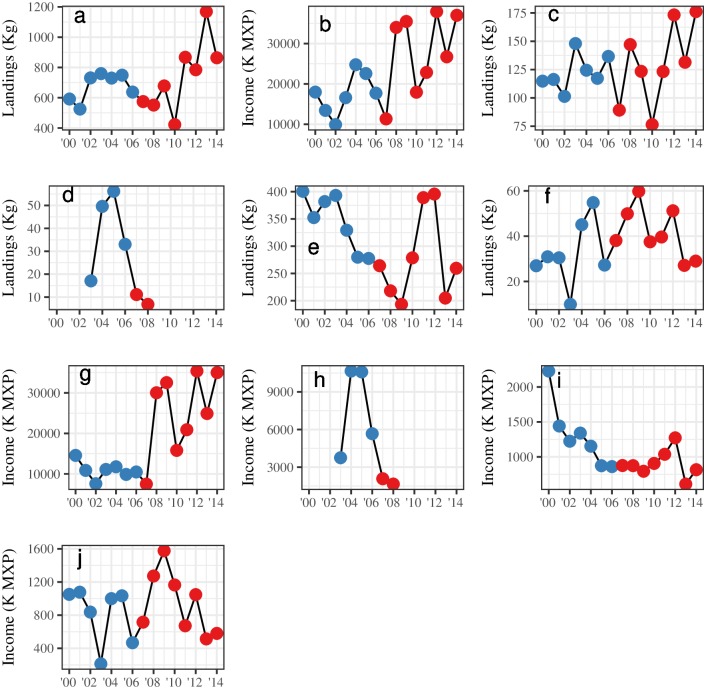
Plots for values of each socioeconomic indicator (y-axis) through time (x-axis). Red and blue correspond to before and after the implementation of the reserve, respectively. This figure contains information for total landings (a), total income (b), lobster landings (c), urchin landings (d), snail landings (e), sea cucumber landings (f), lobster income (g), urchin income (h), snail income (i), and sea cucumber income (j).

One of the main objectives of this reserve was to increase landings. Results of the socioeconomic indicators show that total landings were, on average, 64.20 metric tonnes higher (*p* > 0.1) after the implementation of the reserves, though this cannot necessarily be interpreted as causal, because it relies entirely on a before-after comparison. Total income was $10,344.85 (*p* < 0.05) thousands of Mexican Pesos (K MXP) higher after the implementation of the reserves. On average, lobster and sea cucumber landings increased, while urchin and snail landings and income decreased. Fig 6 presents the changes in these indicators through time, and Table 4 summarizes these results.

**Table 4:** Summary of differences in socioeconomic indicators before and after the implementation of the reserve.

Indicator	Estimate (SD)	t-score
Landings	64.20 (90.07)	0.7127
Income	10344.85 (3982.20)**	2.5978
Lobster landings	7.37 (13.95)	0.5281
Urchin landings	-30.00 (9.49)**	-3.1620
Snail landings	-69.53 (33.82)*	-2.0561
Cucumber landings	9.34 (6.72)	1.3906
Lobster income	14372.85 (3634.64)**	3.9544
Urchin income	-5800.46 (1867.50)**	-3.1060
Snail income	-404.85 (187.07)**	-2.1641
Cucumber income	131.49 (185.66)	0.7082

* Indicate significance level, with (*) indicating p < 0.1 and (**) p < 0.05.

Recall that the governance objectives are evaluated based on the institutions present, not on a specific quantitative linkage between governance and biological or economic outcomes. Data for this reserve suggest that the community is strongly organized, which is a likely driver of the successes reported above [62]. The first point of success is the existence of a fishing cooperative that is also affiliated with a regional federation of cooperatives. These polycentric governance structures allow various levels of organization that have been shown to foster communication and cooperation [50, 57]; federations also provide bargain power with governments [50, 63]. Access to fishing resources is managed through a TURF, permits, and fishing quotas (for some species). It has been suggested that TURFs promotes a sense of stewardship of resources and incentivizes sustainable management [56]. Together, these structures enabled a participative, bottom–up process during the reserve design phase; opinions of all fishing members –and often non-fishing community members– were included. Participation of community members in reserve surveillance and yearly monitoring indicate commitment and interest, and allow informal communication of results to uninvolved community members. Furthermore, the reserve is partially isolated from poaching activity, and fishers have internal regulations pertaining to the reserves. The low level of illegal fishing by members of the community and outsiders both inside and outside the reserve is another indication of effectiveness. Governance indicators are summarized in Table 5.

**Table 5:** Summary of governance indicators.

Indicator	Description
Access to the fishery	Permits, Territorial Use Rights for Fisheries, Quotas (for some fisheries)
Number of fishers	Stable
Legal recognition of reserve	Not recognized
Reserve type	Community-based Marine Reserve
Illegal harvesting	Due to its relative isolations, neither the reserve or TURF suffer from significant illegal harvesting
Management plan	The reserve does not have a management plan, but written rules exist within the cooperative
Reserve enforcement	Fishers have two land stations equipped with radars and patrol boats 24/7 to patrol the reserves.
Size of reserve	The reserve is big enough to protect the targeted sessile or not highly mobile invertebrates (lobster, urchin, snail, cucumber, and abalone)
Reasoning for reserve location	The reserves were put in place in zones that, according to local knowledge, were once very productive. Habitat heterogeneity and ease of monitoring, surveillance and enforcement were also considered.
Membership to fisher organizations	The fishers are part of fisher organizations.
Type of fisheries organizations	The fishers are part of a cooperative (S.C.P.P. Buzos y Pescadores de la Baja California) and are affiliated to a federation (FEDECOOP).
Representation	Reserves were designed by fishers in a bottom-up approach, incorporating expertise from academics and CSO members. This was a highly inclusive and participatory process.
Internal Regulation	Fishers have stringent internal regulations to control fishing effort throughout their TURF, assigning different fishing zones and gears to different teams. Rules pertaining the marine reserves also exist.
Perceived Effectiveness	The fishers have a positive perception about the effectiveness of their reserve, often stating that they have seen significant economic benefits.
Social Impact of Reserve	The reserves have had a significant positive social impact. Fishers are proud to be an example of successgul marine conservation, allowing them to have increased social capital.

## Discussion

We have developed and presented an automated approach for evaluating the effectiveness of marine reserves in Mexico, and perhaps around the world. Here we highlight MAREA’s utility for evidence-based management, and comment on a few of its shortcomings. The findings from Isla Natividad are used purely to validate the relevance of MAREA rather than to discuss particularities of the marine reserve effectiveness, which has been described before [8, 10, 43]. We use examples from the case study to build on the utility of MAREA and discuss ways in which results can be interpreted to inform management.

The causal inference techniques used by MAREA have been suggested [38, 47] and used [21] before in other ad hoc studies. This approach reduces ambiguity in the interpretation of results. For example, invertebrate density decreased through time inside and outside of the reserve (Fig 5h). In this case, a before–after evaluation of the reserve (*i.e.* ignoring the control site) would have incorrectly concluded that the reserve failed to protect invertebrates. On the other hand, a control–impact approach (*i.e.* compare reserve vs. control site only in 2016) would have identified higher densities inside the reserve, concluding that the reserve increases invertebrate density. However, by executing a formal difference-in-differences approach for causal inference, MAREA identifies the changes through time and across sites, and estimates the effect of the reserve on density at *β*_2_ = 91.21 (*p* < 0.05). This approach reveals that invertebrate densities decrease in both sites through time, but the decrease is faster for the control site, thus yielding a positive value for *β*_2_.

The approach used by MAREA to estimate the effect of the reserve on biological indicators requires cautious interpretation of the results. The value of the *β*_2_ coefficient represents the difference between the temporal trends of the reserve and control sites [21]. As exemplified by the case of invertebrate densities, a positive value (*i.e.* *β*_2_ > 0) does not necessarily indicate an increase in the indicator through time, but rather a positive difference with respect to the temporal trend of the control site. The inverse occurs for negative values of *β*_2_.

MAREA provides in-depth analysis and a convenient snapshot overview of the effect of the reserve, allowing users to rapidly identify trends. However, users must interpret multiple indicators at a time to better understand the results. For example, with additional knowledge of local environmental variability (*i.e.* indicator B5: Natural Disturbance), we can better understand the trends in invertebrate densities. As reported before [8], hypoxic conditions that have occurred in Isla Natividad can cause decreases in invertebrate densities, and reserves buffer the negative effect. While MAREA automates the analysis and makes results replicable, proper interpretation will still depend on the user. Results produced by MAREA can only aid in management and decision making when results have been correctly interpreted.

Socioeconomic and governance indicators typically lack a control site, which impede us from using the causal inference techniques employed to measure biological changes [25]. However, we can still extract useful information from them. Again, by combining results from multiple indicators, MAREA can provide insights into the effect of the reserve. For example, lobster and sea cucumber have shown increases in densities, landings, and income. We cannot conclude that landings and income from these species have increased due to the reserve, but we can at least conclude that landings have not decreased. While further information on market behavior of each fishery is needed, these results provide insights into the state of the reserve and its associated fisheries.

As for the governance information, it is difficult to establish causal links between the state of the reserve and the governance structures present in the community. However, providing a single platform (*i.e.* scorecard) or document (*i.e.* technical report) where biological, socioeconomic, and governance information is comprehensively included can aid in management. By using MAREA, this information will be reported across reserves in a standardized way, and can help managers identify overarching patterns across sites.

By making results straightforward to interpret, MAREA may also assist in communication with a broader stakeholder community. While stakeholder involvement in the design and implementation phases of marine reserves is important, that may not be sufficient for ensuring long-term buy-in or success. The scorecard is easily understandable by experts and non-experts, and can be used as an effective tool for communicating the results of annual evaluations. Additionally, the technical report can serve as a tool for managers and scientists to rapidly produce and communicate information at a more technical level.

We recognize that the seven objectives and 29 indicators used by MAREA might not fully describe a reserve in countries other than Mexico. In order to ensure the applicability of the tool to reserves in other countries, further testing in other regions should take place. However, the proposed objectives and indicators provide a starting point to perform the evaluation, to which managers and users can add other indicators (*e.g.* larval dispersal or connectivity) that are relevant to their reserve. Furthermore, MAREA’s value is that it provides a free, simple, and replicable way to perform rigorous impact analysis. The tool can easily be used by fishers, CSO members, and managers in government agencies, providing transparency of the analysis and results. In addition, it can empower and enable local managers and fishers to respond to local change and adapt by allowing direct and easy access to the information.

An evident limitation of MAREA is its dependence on data obtained through a BACI design, and the amount of samples needed to estimate coefficients in Eq (1). It is not uncommon for control sites or baselines to be absent. Properly designing marine reserves by identifying control sites and establishing a baseline before the implementation of the reserve is enough to overcome this issue; reserves for which there is no control site and baseline cannot be evaluated with MAREA. Typical underwater surveys require that at least 12—16 transects are performed for each site (*i.e.* reserve and control) each year. This provides at least 48 samples (12 samples per site, per year), enough to avoid overfitting Eq (1). However, these problems can be easily avoided during the design and implementation phases by anticipating what data will be needed in the eventual evaluation.

To the best of our knowledge, MAREA is the first tool designed to evaluate marine reserves. Previous work [23, 25] addressed MPA evaluation and provided the foundation for our contribution. However, these did not intended to create user-friendly tools to aid in the evaluation. Conservation management tools that automatize complex calculations can have an important impact in management [64]. The use of open data science enables the creation of open-access tools that can address technical gaps and inprove management [28].

The effectiveness of marine reserves continues to be a matter of debate [11, 44, 65]. With current targets set to increase ocean protection, it is important that we understand the effects of our interventions [38] so we can better inform management [47]. It is therefore important that academics, managers, fishers, and CSOs have access to open access tools like MAREA. This is particularly relevant for Mexico and other Latin American countries, where management agencies are often understaffed and underfunded [66], or where materials are often not available in their language. In this context, MAREA provides a simple and replicable way to align management objectives with performance indicators. The proposed methodologies, especially the way in which biological indicators are evaluated, provide valuable information for managers. We acknowledge there is room for improvement in the way in which socioeconomic and governance data are analyzed. Despite this, providing a unifying platform where all indicators can be analyzed and comprehensively presented represents a valuable step towards effective evidence-–based management [47].

The first release of MAREA is now available, and it will continue to be developed and maintained to keep up to date with the literature. This process will incorporate new features, and enhance current ones, aiming to improve user experience and expand the scope of the analysis. Other modifications may also include addition of more objectives and indicators to ensure applicability in other regions, full translation into other languages to avoid any ambiguities introduced via the automatic translation, or reporting effects over time in percentages to aid interpretation. Yet, we believe that this first release represents a major step towards effective, replicable evaluation and management of marine reserves.

## Supporting information

S1 Appendix Survey to collect governance information from fishing communities. English version.
(PDF)

S2 Appendix Survey to collect governance information from fishing communities. Spanish version.
(PDF)

S1 Table Assigned values and reasoning of socioeconomic and governance indicators used to color-code the scorecard in MAREA. (PDF)
