# Supplementary material for: A user-friendly tool to evaluate the effectiveness of no-take marine reserves
Source: PLoS One. 2018 Jan 30;13(1):e0191821. doi: 10.1371/journal.pone.0191821 (PMC5790253; doi:10.1371/journal.pone.0191821)
Supplement: S1 Appendix — English version. (PDF) [file pone.0191821.s001.pdf]

SURVEY TO UNDERSTAND COMMUNITY PERCEPTION OF NO-TAKE MARINE RESERVES

This research is being conducted by TURFeffect, a group of master's students at the Bren School of Environmental Science & Management at the University of California, Santa Barbara, located in the United States of America.
The group is working with Comunidad y Biodiversidad A.C. (COBI) to create a framework for evaluating the success of no-take marine reserves in Mexico. The purpose of this survey is to fill information gaps about the communities that COBI has been working with the past 15 years. This information will be used to perform sample analyses on selected indicators that measure changes in the environment, economy and social structures after the implementation of no-take zones.
The participation at this survey is voluntary and the interviewee may decline to participate or withdraw from participating at any time, without any penalization.

Interviewer: ____________________________________ **Date:** __________________

Name of the community: __________________________________

SECTION 1

The following questions are for demographics purposes only.

Interviewee: ____________________________________ **Gender:** __________ **Age:** ____

Occupation: ____

How long have you lived in this community? ___________________________

**Level of education**	**Diver**
__No formal education	__Yes
__Primary school	__No
__Secondary school	
__Professional school	
__College (Bachelor's degree)	
__Other:____	

How long have you had that job? ___________________________________

How often do you work? ____

If the interviewee is a leader of the fishing community, proceed with the survey in <u>Section 2</u>; if the interviewee is a regular fisher/diver, jump to <u>Section 3</u>

SECTION 2

The following questions should be answered only by leaders of fishing community.

1. Are the fishers of this community organized? If so, how? How many of each type of organization?
__ Cooperative
__ Association
__ Union
__ Other. Please specify:

2. Could you give us the following information about the no-take zones in this area?

Name of the area	Year of implementation	Reasoning for location

3. Who initiated the process of creating the no-take zones? Select all that apply and highlight the top 3 actors.
__ Community
__ Cooperative
__ Non-Governmental Organizations: ____________________
__ Academics
__ Government Agencies: _____________________________
__ Other. Please specify: ______________________________

4. Who is involved in the management, monitoring and enforcement of the no-take zone? Select all that apply.

	**Management**	**Monitoring**	**Enforcement**
Community			
Fishing Community			
Cooperative			
Non-Governmental Organizations:			
Contracted companies:			
Academics			
Government Agencies			
Other. Please specify:			
Who else should participate in these processes?			

5. Are the no-take zones legally recognized by the government?
__ Yes
__ No

5.1. If they are not recognized, has the community already started the process to get its no-take zones recognized?

5.2. If the no-take zone is not recognized and the community did not start the process yet, are they willing to do it?

6. Is there a document or guide that explicitly presents the information about the management of the non-fishing zone?
__ Yes
__ No
__ I don't know

7. How are the local fisheries managed? Select all that apply.
__ TURF: ____
__ Permits: ____
__ Quotas (whole fishery): ____
__ Individual Quota: ____
__ Open Access: ____
__ Other. Please specify: ____

8. How is the enforcement done? Select all that apply
__ Sighting from land
__ Patrol boats
__ VMS (Vessel Monitoring System)
__ Other: Please specify.

9. Does your organization have rules about when, how or where to fish, that does not comes from CONAPESCA or other government agencies?
__ Yes
__ No
__ I don't know

	**Before/at the implementation**	**Nowadays**	**Reason**
**10**. Number of fishers in the community			

11. Would you say that an unusual natural event of "great proportions", such as a hurricane, El Niño, hypoxia or related events, occurred in the area of the reserve since the last monitoring event? If yes, what was the event?

____

SECTION 3

If the interviewee is just a Fisher or other person involved with fisheries (e.g. fish buyer or seller), then the survey should start here.

12. Who participates on the decisions for the no-take zones? Select all that apply.
__ Community
__ Fishing Community
__ Cooperative
__ Non-Governmental Organizations: ___________________
__ Academics
__ Government Agencies.: ___________________
__ Others. Please, specify: ___________________

12.1 Do you thinks someone else should be involved in the decision making process? Who else should participate in these processes?

13. How well do you think the vigilance and enforcement works in this community?
__ Very good
__ Good
__ Moderate
__ Bad
__ Very Bad

14. From 0 to 5, how would you rate the illegal fishing (by illegal gear, effort, area) inside your fishing area? 0=low, 5=high

	By people from the community	By people from outside the community
Inside the NTZs		
Outside the NTZs		

15. After the implementation of the reserve, has government enforcement:
__ Increased
__ Stayed the same
__ Decreased

16. Enforcement works better when the government participates.
__ Strongly agree
__ Agree
__ Not sure
__ Disagree
__ Strongly disagree

17. Enforcement works better when the fishing group participates.
__ Strongly agree
__ Agree
__ Not sure
__ Disagree
__ Strongly disagree

	**Before/at the implementation**	**Nowadays**	**Reason**
**18.** How would you rate the level of alternative economic opportunities?	__ Very High<br>__ High<br>__ Moderate<br>__ Restricted<br>__ Low<br>__ Null	__ Very High<br>__ High<br>__ Moderate<br>__ Restricted<br>__ Low<br>__ Null	
**19.** What is the level of illegal fishing in the region?	__ Very High<br>__ High<br>__ Moderate<br>__ Restricted<br>__ Low<br>__ Null	__ Very High<br>__ High<br>__ Moderate<br>__ Restricted<br>__ Low<br>__ Null	

20. How would you answer the following questions?

I fish inside the NTZs	__Never<br>__Sometimes<br>__Always
Other fishers from the community fish inside the NTZs	__Never<br>__Sometimes<br>__Always
Fishers from nearby communities fish inside the NTZs	__Never<br>__Sometimes<br>__Always

21. What do you think about the implementation of the no-take reserve with respect to:

21.1. The environment

Indicator	Improved	Stable	Worsened	Reason for change
Species richness				
Fish length				
Density				
Biomass				
Abundance of predators				

21.2. The communities' economy

Indicator	Improved	Stable	Worsened	Reason for change
Total landings				
Managed species landings				
Fisheries revenue				
Alternative economic activities				

21.3. Local governance and management

__ Improved
__ Stayed the same
__ Worsened

22. What is the general impact of the reserves in the community?

__ Positive
__ None
__ Negative

23. Do you think the no-take zone is effective?

__ Yes
__ No
__ I don't know

24. Do you think reserves should be eliminated, modified or stay the same?

Zone	Action	Why	How
	__ Eliminate<br>__ Keep the same<br>__ Modify		
	__ Eliminate<br>__ Keep the same<br>__ Modify		
	__ Eliminate<br>__ Keep the same<br>__ Modify		
	__ Eliminate<br>__ Keep the same<br>__ Modify		
	__ Eliminate<br>__ Keep the same<br>__ Modify		

25. Would like to add any comment?
