# Supplementary material for: A user-friendly tool to evaluate the effectiveness of no-take marine reserves
Source: PLoS One. 2018 Jan 30;13(1):e0191821. doi: 10.1371/journal.pone.0191821 (PMC5790253; doi:10.1371/journal.pone.0191821)
Supplement: S2 Appendix — Spanish version. (PDF) [file pone.0191821.s002.pdf]

ENCUESTA PARA ENTENDER LA PERCEPCIÓN DE LA COMUNIDAD SOBRE ZONAS DE NO PESCA

Esta investigación está siendo realizada por TURFeffect, un grupo de estudiantes de maestría de Ciencias Ambientales y Manejo la Escuela Bren en la Universidad de California, Santa Bárbara, ubicada en Estados Unidos de América.

El grupo está trabajando con Comunidad y Biodiversidad A.C. (COBI) para crear un marco de referencia/sistema para evaluar la efectividad de las zonas de no pesca en México. El propósito de esta encuesta es llenar vacíos de información sobre las comunidades con las que COBI ha trabajado en los últimos 15 años. Esta información será usada para realizar evaluar la percepción que los usuarios tienen sobre algunos indicadores biofísicos. Además, recupera información socioeconómica y de gobernanza sobre la comunidad y las zonas de no pesca. La participación en esta encuesta es voluntaria y el entrevistado puede negarse a participar o retirarse de la entrevista en cualquier momento y sin ningún tipo de penalización.

Entrevistador: ______________________________________ **Fecha:** ___________________

Nombre de la comunidad: ____________________________________

SECCIÓN 1

Las siguientes preguntas están enfocadas en la información demográfica.

Entrevistado/a:_____________________________________ **Genero:** __________ **Edad:**____

Titulo/Ocupación: ____

¿Cuántos años ha vivido en esta comunidad? _____________________________

Nivel de educación:
- __ No educación formal
- __ Escuela primaria
- __ Escuela secundaria
- __ Preparatoria
- __ Bachillerato
- __ Otro

Buzo Monitor:
- __Si
- __No

¿Por cuántos años ha tenido este trabajo? ___________________

¿Con qué frecuencia trabaja? __________________

Si el/la entrevistado/a es un/a líder de la comunidad pesquera, continúe con la sección 2 de la entrevista. Si el/la entrevistado/a es un/a pescador/a y/o buceador/a, continúe con la sección 3 de la entrevista.

SECCIÓN 2

Las siguientes preguntas deben ser respondidas únicamente por líderes de la comunidad pesquera.

1. ¿Cuáles y cuántos tipos de organización están presentes en la comunidad pesquera?

__ Cooperativa
__ Asociación
__ Sindicato
__ Comité comunitario
__ Federación
__ Confederación
__ Ningún
__ Otro. Por favor especifique:

2. En la tabla de abajo, llene el nombre de la zona de no pesca, el año de implementación y una breve descripción sobre el razonamiento para establecer esa zona como una zona de no pesca.

Nombre del área	Año de implementación	Por qué fue escogida esta ubicación

3. ¿Quién inició el proceso de creación de las zonas de no pesca? Seleccione todas las respuestas que apliquen

__ Comunidad
__ Comunidad pesquera
__ Cooperativa
__ Organizaciones No Gubernamentales: ______________________
__ Académicos
__ Agencias de Gobierno: ___________________________________
__ Otros. Por favor especifique: ____________________________

4. ¿Quién está involucrado en el manejo, monitoreo y procuración de la zona de no pesca? Marque todas las respuestas que apliquen.

	Manejo	Monitoreo	Procuración
**Comunidad**			
**Comunidad pesquera**			
**Cooperativa**			
**Organizaciones No Gubernamentales:**			
**Empresas contratadas**			
**Académicos**			
**Agencias de Gobierno.:**			
**Otros. Por favor especifique:**			
**¿Quién más debería participar en estos procesos?**			

5. ¿Están reconocidas legalmente las zonas de no pesca?
___ Si
___ No

5.1. Si no están reconocidas, ¿ha comenzado la comunidad el proceso para que las zonas de no pesca sean reconocidas legalmente?

5.2. Si las zonas de no pesca no han sido reconocidas legalmente y no se ha comenzado el proceso aún, ¿está la comunidad dispuesta a hacerlo?

6. ¿Existe un documento o guía en la que se presenten explícitamente la información sobre el manejo de la zona de no pesca?
___ Si
___ No
___ No Sé

7. ¿Cómo se manejan/gestionan las pesquerías locales? Seleccione todas las respuestas que apliquen
__ Derechos de uso territorial (Concesiones): ______________________
__ Permisos: _____________________________
__ Cuotas (toda la pesquería): _____________________________
__ Cuotas Individuales: _____________________________
__ Acceso abierto: _____________________________
__ Otro. Por favor especifique: ____

8. ¿Cómo se realiza la procuración / vigilancia? Seleccione todas las respuestas que apliquen
__ Avistamiento desde tierra
__ Botes patrulla
__ Sistema de localización de embarcación
__ Otros. Por favor especifique:

9. ¿Su organización tiene reglas sobre cuándo, cómo o dónde pescar, que no provienen de CONAPESCA u otras agencias gubernamentales?
__ Si
__ No
__ No Sé

	**Antes de / en la implementación**	**Actualmente**	**Motivo del cambio**
**10.** Número de pescadores en la comunidad			

11. ¿Diría usted que algún evento natural inusual como huracán, El Niño, eventos de hipoxia o similares desde el último monitoreo? ¿De ser así, cuál fue el evento?

___

SECCION 3

Si el entrevistado es únicamente un pescador o está involucrado con la pesquería (ej. Comprador o vendedor de pescado), por favor, inicie la encuesta aquí.

12. ¿Quién participa en la toma de decisiones para la zona de no pesca? Seleccione todas las respuestas que apliquen

__ Comunidad
__ Comunidad pesquera
__ Cooperativa
__ Organizaciones No Gubernamentales: ___________________
__ Académicos
__ Agencias de Gobierno.: ___________________
__ Otros. Por favor especifique:

12.1. ¿Cree que se debería involucrar a alguien más en esta toma de decisiones? ¿A quién?

13. ¿Qué tan bien cree usted que la vigilancia y el cumplimiento de la ley funcionan en su área de pesca?

__ Muy bien
__ Bien
__ Moderadamente
__ Mal
__ Muy mal

14. En una escala de 0 a 5, ¿Cómo calificaría usted la pesca ilegal (por artes y prácticas ilegales) en su área de pesca? 0 = bajo, 5 = alto

	**Por personas del grupo pesquero**	**Por personas de fuera del grupo pesquero**
**Dentro de la(s) zona(s) de no pesca**		
**Fuera de la(s) zona(s) de no pesca**		

15. Después de la implementación de la reserva/zona de no pesca, la procuración/monitoreo por parte del gobierno ha:

__ Incrementado
__ Permanecido igual
__ Disminuido

16. La procuración/monitoreo funciona mejor cuando el gobierno participa.

__ Totalmente de acuerdo
__ De acuerdo
__ No estoy seguro
__ En desacuerdo

__ Completamente en desacuerdo

17. La procuración/monitoreo funciona mejor cuando el grupo pesquero participa.
__ Totalmente de acuerdo
__ De acuerdo
__ No estoy seguro
__ En desacuerdo
__ Completamente en desacuerdo

	**Antes de / en la implementación**	**Actualmente**	**Motivo del cambio**
**18.** ¿Cómo calificaría el número de oportunidades de trabajo alternativas a la pesca?	__ Muy Alto<br>__ Alto<br>__ Moderado<br>__ Restringido<br>__ Bajo<br>__ Nulo	__ Muy Alto<br>__ Alto<br>__ Moderado<br>__ Restringido<br>__ Bajo<br>__ Nulo	
**19.** ¿Cuál es el nivel de pesca ilegal en la región?	__ Muy Alto<br>__ Alto<br>__ Moderado<br>__ Restringido<br>__ Bajo<br>__ Nulo	__ Muy Alto<br>__ Alto<br>__ Moderado<br>__ Restringido<br>__ Bajo<br>__ Nulo	

20. ¿Cómo respondería a los siguientes enunciados?

Yo pesco dentro de las zonas de no pesca.	__ Nunca<br>__ A veces<br>__ Siempre
Otros compañeros de mi comunidad pescan dentro de las zonas de no pesca.	__ Nunca<br>__ A veces<br>__ Siempre
Otros compañeros de otras comunidades cercanas pescan dentro de los refugios.	__ Nunca<br>__ A veces<br>__ Siempre

21. ¿Qué piensa usted acerca de la implementación de las zonas de no pesca con respecto a:
21.1. El medio ambiente

Indicador	Mejor	Igual	Peor	Motivo del cambio
**Número de especies**				
**Longitud de los peces**				
**Densidad de organismos**				
**Biomasa**				
**Abundancia de depredadores**				

21.2. Situación económica de la comunidad pesquera

Indicador	Mejor	Igual	Peor	Motivo del cambio
**Desembarques totales**				
**Desembarque de especies objetivo**				
**Ingresos por la pesca**				

22.3. Gobierno y gestión local. Encierre en un círculo su respuesta
__ Mejor
__ Igual
__ Peor

22. ¿Cuál es el impacto general de las reservas en la comunidad?
__ Positivo
__ Nulo
__ Negativo

23. ¿Cree que las zonas de no pesca sean efectivas?
__ Sí
__ No
__ No sé

24. En su opinión, ¿cree que las reservas deban ser eliminadas, modificadas o mantenidas?

Zona	Acción	¿Por qué?	¿Cómo?
	__ Eliminar<br>__ Mantener<br>__ Modificar		
	__ Eliminar<br>__ Mantener<br>__ Modificar		
	__ Eliminar<br>__ Mantener<br>__ Modificar		
	__ Eliminar<br>__ Mantener<br>__ Modificar		
	__ Eliminar<br>__ Mantener<br>__ Modificar		

25. ¿Le gustaría agregar algún comentario?
