# Supplementary material for: A user-friendly tool to evaluate the effectiveness of no-take marine reserves
Source: PLoS One. 2018 Jan 30;13(1):e0191821. doi: 10.1371/journal.pone.0191821 (PMC5790253; doi:10.1371/journal.pone.0191821)
Supplement: S1 Table — (PDF) [file pone.0191821.s003.pdf]

Indicator	Options	Value	Reasoning	References
Alternative economic opportunities	Increase	1	Having alternative economic opportunities provides users with an alternative source of income and can compensate forgone profits from implementing a reserve. With no alternative economic opportunities, this might be a source of instability for the community.	[1][2]
	Stable / Decrease	-1		
Access to the fishery	TURF	1	TURFs promote stewardship of marine resources	[3][4]
	Permits	1	Fishing permits (or licenses) create entry restrictions, which can limit the total amount of effort applied to the stock	[5]
	Quotas for the whole fishery	1	Total allowable catches and Individual Transferable Quotas cap total catches at a maximum.	[6]
	Individual Quotas	1		
	Open Access	-1	The fishery is unrestricted. This can lead to long-term depletion of stocks.	
Number of fishers	Lower / Stable	1	Maintaining or decreasing the number of fishers (a proxy for fishing effort) might help stabilize fishing effort. An increased number of fishers with reduced fishing ground might lead to an overall decrease in per capita income.	
	Higher	-1		
Legal recognition of reserve	Yes	1	Legal recognition of the reserve increases threat of punishment, which can de-incentivize illegal fishing.	[7]
	No	-1		
Degree of illegal harvesting	Lower	1	Illegal fishing decreases biophysical indicators (e.g. biomass, density), and therefore, socioeconomic indicators (i.e. landings and income from landings).	
	Higher	-1		
Management plan	Yes	1	There is a better chance that members will be aware of the rules and limits of the reserve. Therefore, it is more likely that rules will be obeyed.	
	No	-1	Members will not be aware of the rules and limits of the reserve. Therefore, it is less likely that rules will be obeyed.	
Reserve enforcement	Patrol boats	1	Any means of reserve enforcement is valid. Enforcing your reserve will allow you to keep illegal fishing to the limits.	
	Sighting from land	1		
	AIS	1		
	Government	1		

Indicator	Options	Value	Reasoning	References
	Community	1		
Size of reserve	Smaller than home range	-1	It is important that reserves can match the home range of the target species	[8]
	Same or larger than home range	1		
Membership to fisher organizations	Yes	1	Provides a platform for communication and cooperation. Not belonging to a group might reduce the capacity of cooperation and participation.	[9][10]
	No	-1		
Representation	High	1	A participatory process in which diverse stakeholders are included fosters communication and anticipates possible issues. At the same time, an inclusive process secures buy-in from the users, which can lead to more effective interventions.	[1][2]
	Low	-1		
Internal Regulation	Yes	1	An organization's internal set of rules are often stricter than formally recognized regulations and tailored to meet internal management objectives. Additionally, they tend to have support from all the community and are thoroughly enforced.	
	No	-1		
Perceived Effectiveness	Yes	1	If members of the fisheries organizations perceive that the reserves help them meet stated objectives, then they will be more likely to comply with the rules of the no-take area.	[2]
	No	-1		

References

1. Bennett NJ, Dearden P (2014) Why local people do not support conservation: Community perceptions of marine protected area livelihood impacts, governance and management in Thailand. Marine Policy 44: 107–116. doi:10.1016/j.marpol.2013.08.017.

2. Alcala AC, Russ GR (2006) No-take Marine Reserves and Reef Fisheries Management in the Philippines: A New People Power Revolution. AMBIO: A Journal of the Human Environment 35: 245–254. doi:10.1579/05-A-054R1.1.

3. Afflerbach JC, Lester SE, Dougherty DT, Poon SE (2014) A global survey of "TURF-reserves", Territorial Use Rights for Fisheries coupled with marine reserves. Global Ecology and Conservation 2: 97–106. doi:10.1016/j.gecco.2014.08.001.

4. McCay B (2017) Territorial use rights in fisheries of the northern Pacific coast of Mexico. BMS 93: 69–81. doi:10.5343/bms.2015.1091.

5. Townsend RE (1990) Entry restrictions in the fishery: A survey of the evidence. Land Econ 66: 359. doi:10.2307/3146619.

6. Costello C, Gaines SD, Lynham J (2008) Can catch shares prevent fisheries collapse? Science 321: 1678–1681. doi:10.1126/science.1159478.

7. Di Franco A, Thiriet P, Di Carlo G, Dimitriadis C, Francour P, et al. (2016) Five key attributes can increase marine protected areas performance for small-scale fisheries management. Sci Rep 6: 38135. doi:10.1038/srep38135.

8. Edgar GJ, Stuart-Smith RD, Willis TJ, Kininmonth S, Baker SC, et al. (2014) Global conservation outcomes depend on marine protected areas with five key features. Nature 506: 216–220. doi:10.1038/nature13022.

9. Espinosa-Romero MJ, Rodriguez LF, Weaver AH, Villanueva-Aznar C, Torre J (2014) The changing role of NGOs in Mexican small-scale fisheries: From environmental conservation to multi-scale governance. Marine Policy 50: 290–299. doi:10.1016/j.marpol.2014.07.005.

10. McCay BJ, Micheli F, Ponce-Díaz G, Murray G, Shester G, et al. (2014) Cooperatives, concessions, and co-management on the Pacific coast of Mexico. Marine Policy 44: 49–59. doi:10.1016/j.marpol.2013.08.001.
